# Targets and study design for symptom-focused trials aimed at patients with cirrhosis: An expert consensus

**DOI:** 10.1097/HC9.0000000000000135

**Published:** 2023-06-02

**Authors:** Arpan A. Patel, Elliot B. Tapper, Fasiha Kanwal, Christopher D. Woodrell, Lissi Hansen, Jennifer C. Lai, Shari Rogal, Cara McDermott, Mina Rakoski, Nneka N. Ufere

**Affiliations:** 1 Tamar and Vatche Manoukian Division of Digestive Diseases, University of California, Los Angeles, Los Angeles, California, USA; 2 VA Greater Los Angeles Healthcare System, Los Angeles, California, USA; 3 Division of Gastroenterology and Hepatology, University of Michigan, Ann Arbor, Michigan, USA; 4 Baylor College of Medicine and Michael E DeBakey VA Medical Center, Houston, Texas, USA; 5 Brookdale Department of Geriatrics and Palliative Medicine, Icahn School of Medicine at Mount Sinai, New York, New York, USA; 6 Geriatric Research, Education and Clinical Center, James J. Peters VA Medical Center, Bronx, New York, USA; 7 Oregon Health & Science University, School of Nursing, Portland, Oregon, USA; 8 Division of Gastroenterology and Hepatology, University of California, San Francisco, California, USA; 9 Center for Health Equity Research and Promotion, VA Pittsburgh Healthcare System, Pittsburgh, Pennsylvania, USA; 10 Division of Gastroenterology, Hepatology, and Nutrition, University of Pittsburgh, Pittsburgh, Pennsylvania, USA; 11 Division of Geriatrics, Department of Medicine, Duke University, Durham, New Carolina, USA; 12 Geriatric Research, Education and Clinical Center, Durham VA Medical Center, Durham, New Carolina, USA; 13 Division of Gastroenterology and Hepatology, Loma Linda University Health, Loma Linda, California, USA; 14 Gastrointestinal Unit, Department of Medicine, Massachusetts General Hospital, Harvard Medical School, Boston, Massachussetts, USA

## Abstract

Symptom-focused trials are critically needed for patients with cirrhosis. However, this work would benefit from standard processes and validated measures.

**Methods:**

A writing group was formed among hepatologists, nurses, palliative care providers, pharmacists, and clinical trial experts focused on symptom management in patients with cirrhosis to define the key (1) components of trial design, (2) symptom targets, (3) measurement, and (4) outcomes for each target. From July 2022 to January 2023, panelists participated in an iterative process of developing and arriving at a consensus for each component. The goal was to provide consensus definitions that can be operationalized in future clinical trials, including for patients with cirrhosis.

**Results:**

The panel reached a consensus on key reporting features for clinical trials, along with considerations for study design. Nine key symptom targets (muscle cramps, pruritus, pain, fatigue, sexual dysfunction, sleep disorders, depression and anxiety, nausea/vomiting, and dyspnea/breathlessness) were identified. The panel selected instruments that can be considered for clinical trials based on psychometric validation and previous experience. The panel identified ongoing needs, including instrument validation, safety data, evidence about non-pharmacologic interventions, and comparative effectiveness studies.

**Conclusion:**

This expert panel identified key design, reporting, and measurement elements to standardize processes and measures in future symptom-focused clinical trials in the context of cirrhosis.

## Graphical abstract

**Figure:**
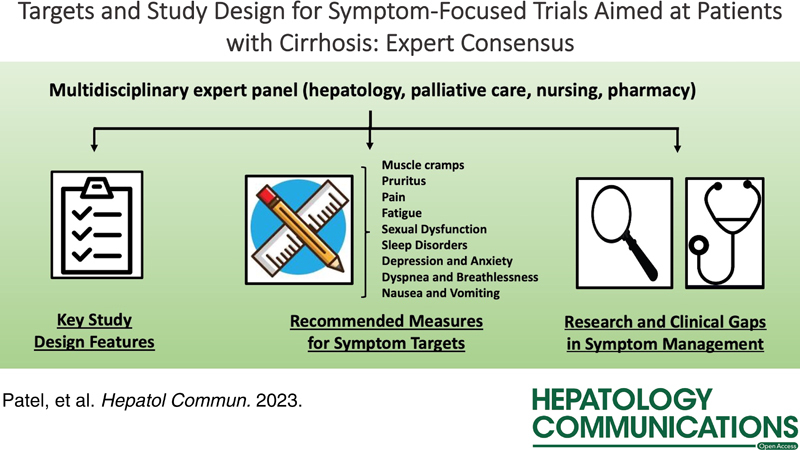

## INTRODUCTION

Cirrhosis is a serious illness characterized by high mortality, unpredictable illness trajectories, and a high burden of physical and psychological symptoms.^1^ Conventional management of symptoms in this population is oriented toward treating decompensation events (eg, ascites, and HE). However, most people living with cirrhosis experience multiple additional distressing symptoms common among patients with serious illness.^2^–^4^ Symptoms such as muscle cramps, itching, disordered sleep, chronic pain, and depression are highly prevalent and degrade the quality of life and functioning. Despite their prevalence and impact, however, these symptoms are under-addressed both clinically and by research, leading to an absence of high-quality data regarding treatment approaches. Efforts to improve research in symptom management are beset by 2 mounting unmet needs: (1) lack of standardization of clinical trial designs and (2) limited consensus about priority symptoms and their measurement. We sought to close these gaps using an iterative process with this expert consensus guidance statement.

## METHODS

### Participants

A core writing group (A.P., E.T., and N.N.U.) identified potential expert group members, with expertise in palliative and supportive care and clinical trial development, as evidenced by a previous track record of publications, including serving on writing groups for clinical practice guidelines, and ongoing research. A multidisciplinary expert panel composed of 10 clinicians from hepatology, palliative care, nursing, and pharmacy who represented geographic and practice setting diversity was selected and convened to establish a framework for designing symptom-focused studies in cirrhosis.

### Aims

The goals of this project were to synthesize expert recommendations highlighting key considerations in study design, symptom targets for interventions, and a proposed toolbox of measures for symptom assessment.

### Framework

The framework for study execution was adapted from the position paper of LiverHope Consortium on methodological aspects of clinical trial design for patients with decompensated cirrhosis and expanded with all authors contributing.^5^ Briefly, this framework posited recommended processes on designing and reporting results of cirrhosis clinical trials investigating disease-modifying therapies, including defining appropriate endpoints and outcomes assessments. Our project aimed to address the lack of standardization of clinical trial designs by applying the LiverHope framework to symptom-focused clinical trials.

### Approach

#### Phase 1

The core writing group (A.P., N.U., and E.T.) drafted skeleton tables for each of the project goals. This included draft versions of Table 1 (reporting recommendations for symptom management trials in cirrhosis), Table 2 (common study features and their requirements), and Table 3 (commonly used measures for key symptom targets in cirrhosis and tested treatment).

**TABLE 1:** Reporting recommendations for symptom management trials in cirrhosis

Setting and location	Specify the location of recruitment (eg, community setting, ambulatory clinic, inpatient) and follow-up
Baseline characteristics	Demographics, etiology of liver disease, alcohol and substance use disorder status, insurance status, and some measures of social support and socioeconomic status Child-Pugh Classification, MELD-Na score, history of ascites, history of HE, history of SBP, paracentesis requirement, TIPS-in-situ, diuretic use, lactulose use, rifaximin use, and beta-blocker use Extrahepatic comorbidities, functional status, mental health comorbidities CNS-active medications, including pain medications, antidepressants, antianxiety medications, antipsychotics, antiepileptics All adjunctive therapies used to address the symptom under study, including treatment of secondary causes List of restricted and prohibited medications during the treatment period
Endpoints	Primary, secondary, and exploratory endpoints should be defined Study duration should be appropriate to detect differences in the outcomes selected Report absolute differences in outcomes and, when possible, the proportions achieving response, along with the number not completing the trial due to adverse events, mortality, and transplant Report MCID for PRO or if not standardized, then provide justification for that MCID and consideration of a clinical anchor Justification for the use of a PRO instrument as the primary outcome, including evidence of its measurement properties, scoring system, and MCID if available
Statistics	A statistical analysis plan should be developed before starting the trial with the timing of expected analyses, stopping rules, and proposed methods of handling missing data An *a priori* sample size calculation is required, ideally using criteria for clinical significance (eg, MCID, responder definition) when known if PRO is the primary outcome or endpoint Consider the need for competing-risk and time-dependent analyses Consider the need to account for clustering in multicenter data
Safety data	Adverse event data should be included. Liver-related events should be specified. COVID-related events can be included
Ethical considerations	Study conducted under appropriate IRB oversight Consent process and compensation described Consideration of whether/how those without the capacity to consent are included Consideration of whether clinical equipoise exists between study arms
Recruitment/enrollment	Inclusion of CONSORT flow diagram Collection of baseline PRO data before randomization and clinical assessments to reduce bias Plans for PRO assessment of patients who withdraw early from the study
Other reporting	Blinding procedures Protocol fidelity monitoring Patient or proxy reporting Data collection plan outlining how, when, and where PRO data will be collected from study participants

Abbreviations: MELD-Na, Model for End-stage Liver Disease-Sodium; SBP, spontaneous bacterial peritonitis; CNS, Central nervous system; MCID, minimal clinically important difference, PRO: patient-reported outcome; IRB, institutional review board; CONSORT, Consolidated Standards of Reporting Trials.

**TABLE 2:** Common study features and their requirements

Target population	Inclusion criteria	Cirrhosis should be defined using standard clinical, imaging, and histological criteria.
		No upper age limits (for adults) or limits on language, education, and/or race/ethnicity
		Minimal symptom burden criteria should be established for the symptom addressed
	Exclusion criteria	Rationale for inclusion/exclusion of decompensated cirrhosis
		Unable to consent due to active encephalopathy or dementia
		All exclusions explicitly justified
Study Design	Study arms	Rationale for the use of a single-arm vs. two-arm (placebo control vs. active control) vs. cross-over study
		If randomization is employed, rationale for the use of stratification based on symptom severity, disease severity, or concomitant therapies should be specified
	Control Groups	Appropriate choice and rationale for the control group: placebo control, attention control (behavioral interventions), “best supportive care” (behavioral interventions)
Intervention	Dosing	Rationale for dose selection (prior safety, pharmacokinetic, preliminary efficacy data) for pharmacologic interventions
		Rationale for dose adjustments as needed for concomitant conditions or medications
		Rationale for intervention delivery (number/frequency/duration of the session) for behavioral interventions
	Other considerations	If a washout period is employed, the duration should be specified as the minimum necessary amount of time, and the timing with respect to the intervention should be specified with its rationale
		Postprotocol therapies should be specified
		Rescue therapies should be specified
Endpoints	Pilot trials	Feasibility outcomes should include the proportion of candidates enrolled, the proportion of enrolled completing the study, reasons for dropout, adherence to the intervention, and dose achieved; acceptability of the intervention should be measured
		Therapeutic effects can be evaluated as exploratory aims
	Phase II-III	Time to the primary endpoint should be tailored to the severity of the underlying liver disease but long enough to assess the efficacy of first-line therapy
		Validated instruments (see **Endpoint Assessment** section), ideally patient-reported outcomes, should be prioritized as primary endpoints
		The primary and secondary endpoints should specify the specific features of the symptoms under investigation (eg, intensity, frequency, duration, interference with specified domains of HRQOL)

Abbreviation: HRQOL, Health-related quality of life

**TABLE 3:** Commonly-used measures for key symptom targets in cirrhosis and tested treatment.

Symptoms	Pathophysiology	Studied treatments (using randomized controlled trial design)
Muscle cramps	Alterations in nerve function, energy metabolism, and plasma volume	Effective: taurine,^6^ branched-chain amino acids,^7^ quinidine,^8^ pickle juice^9^
Pruritus	Unclear; possibly due to dysregulation of bile acids or lysophosphatidic acid accumulation	Effective*: cholesytramine,^10^ rifampin,^11^ naltrexone,^12^ sertraline,^13^ bezafibrate^14^ Not effective: colesevelam,^15^ gabapentin^16^
Neuropathic pain	Somatosensory pain that can occur in patients with peripheral neuropathy associated with cirrhosis	None
Nociceptive pain	May be related to somatic and visceral pain secondary to ascites, splenomegaly, cramps, musculoskeletal complaints, mastalgia, or fractures	None
Nociplastic pain	Alterations in peripheral nociceptors, which may be associated with fibromyalgia	None
Fatigue	Central fatigue may be due to changes in neurotransmission, whereas peripheral fatigue may be associated with neuromuscular dysfunction from fatigue/muscle wasting; other contributing factors including hypothyroidism, vitamin D deficiency, depression, adrenal insufficiency, anemia, and medication side effects	Not effective: fluvoxamine,^17^ modafanil^18^
Sexual dysfunction	Main categories: (1) erectile dysfunction; (2) reduced libido; and 3) hypogonadism; causes can be hypothalamic/pituitary suppression, primary testicular dysfunction, high estrogen: testosterone ratio, medications, autonomic neuropathy, mental health disorders, or physical limitations (like ascites)	Effective: tadalafil^19^
Sleep disorders	Include insomnia, poor sleep quality, excessive daytime sleepiness, and sleep-wake inversion; can be associated with liver disease symptoms and diagnoses as well (HE, ascites/edema, pruritus, sleep apnea, NAFLD)	Effective: melatonin,^20^ zolpidem,^21^ hydroxyzine^22^
Depression and anxiety	If not primary, it may be related to secondary causes, such as vitamin deficiency, encephalopathy, and dementia.	None
Nausea/Vomiting	May be associated with ascites burden, medications, adrenal insufficiency, electrolyte imbalance, uremia, reflux, constipation, or gastroparesis	None
Dyspnea/Breathlessness	Portal hypertension	None

^A^all studied in cholestatic liver disease patients.

#### Phase 2

The components of each table were reviewed and edited by all authors. Authors adapted the LiverHope framework for symptom-focused clinical trials by providing suggestions for additional design domains germane to our focus (Tables 1 and 2). Each author independently added the symptoms, which they perceived were important to address in studies of patients with cirrhosis (Table 3).

#### Phase 3

Using a RedCap survey, each author then provided guidance on appropriate measures for each symptom listed in Table 3. Guidance was provided to select measures prioritized based on a predefined hierarchical scheme, including (1) validated for use in cirrhosis, (2) have been employed in randomized trials enrolling participants with cirrhosis (3) used in single-arm studies of patients with cirrhosis, or (4) instruments validated in other populations with proven responsiveness to interventions and without significant floor or ceiling effects. Authors were instructed to provide key references supporting their selections. The results of the survey were synthesized. When possible, the inclusion of widely recommended indices from other consensus documents (ie, the NIH Clinical Pain Management program^23^) was considered. A draft Table 4 (key symptom targets, measures, and psychometric properties) was then sent to the authors for comment.

**TABLE 4:** Key symptom targets, measures, and psychometric properties

Symptom	Outcome	Measure	Prior use in cirrhosis	MCID or MDC [population used to establish it]
Muscle cramps	Cramp intensity	VAS	RCT^6^,^9^	No reported MCID or MDC
	Cramp frequency	Cramps per week (self-report)	RCT^6^,^8^,^9^	No reported MCID or MDC
	Cramp duration	Minutes (self-report)	RCT^6^,^9^	No reported MCID or MDC
Pruritus	Pruritus intensity	VAS^24^	RCT^11^–^16^,^25^	MCID: 2–3 points (out of 10) [192 patients with chronic itch and non-cirrhotic]^26^
	Composite (degree, duration, direction, disability, and distribution)	5-D Pruritus Scale^27^	RCT^28^ Single-arm trial^29^,^30^	Original validation cohort (n=234) included 63 (27%) liver disease patients^27^ No reported MCID or MDC
Nociceptive Pain (or Unspecific Pain)	Pain intensity, Severity, and Interference	Brief pain inventory^31^	Observational^32^	MCID: ~2 point change (out of 10) [1411 patients with fibromyalgia]^33^
	—	Pain, enjoyment of life and General Activity Scale (PEG)^34^	None	MDC: Standard error of measurement of 1.8–1.9 [427 adults with chronic musculoskeletal pain]^35^
	—	Pain Disability Index^36^	Observational^37^	MCID: 8.5–9.5 points (out of 70) [242 patients with chronic back pain]^38^
	—	McGill Pain Questionnaire^39^	Observational^37^	MCID: 1–2.3 points (out of 5) [114 patients receiving spinal cord stimulation for failed back surgery syndrome]^40^
	Pain interference only	PROMIS Pain Interference^41^	Observational^42^	MCID: 3.5–5.5 points [414 patients with low back pain]^43^ and 4–6 points [101 patients with advanced-stage cancer]^44^ on the T-score scale
Neuropathic Pain	Neuropathic pain characteristics	PainDETECT^45^	None	No reported MCID or MDC
	—	Neuropathic Pain Symptom Inventory^46^	None	No reported MCID or MDC
	—	Neuropathic Pain Scale^47^	None	No reported MCID or MDC
Nociplastic Pain	Presence of pain	Fibromyalgia Survey Questionnaire (FSQ)^48^	None	No reported MCID or MDC
Fatigue	Presence, severity, and impact of fatigue	The Fisk Fatigue Severity Score (FFIS)^49^	RCT^17^,^18^,^50^,^51^	Validated in a cohort of 58 patients with primary biliary cholangitis^52^ MCID: 10–20 points (out of 160) [184 patients with multiple sclerosis]^53^
	—	Multidimensional Fatigue Inventory^54^	RCT^17^	MCID: 11.5–13.3 points (global change) and 6.8–9.6 points (improvement) (out of 100) [141 patients with lupus or rheumatoid arthritis]^55^
	—	PROMIS-fatigue short form^56^	None	MCID: 2–3 points (out of 35) [101 patients with cancer]^55^
Sexual Dysfunction	Male sexual function Only	International Index of Erectile Function (IIEF)^57^	RCT^19^	MCID: 4 points (out of 25) [1240 men with erectile dysfunction enrolled in tadalafil clinical trials]^58^
	Female sexual function Only	Female Sexual Function Index (FSFI)^59^	Observational^60^	MCID: 0.5–1.0 points (out of 6) for each FSFI domain [108 women with sexual dysfunction]^61^
	Sexual function (both sexes)	Arizona Sexual Experience Scale^62^	Observational^63^	No reported MCID or MDC
		PROMIS Sexual Function^64^	None	No reported MCID or MDC
Sleep Disorders	Sleep quality and disturbance	Pittsburgh Sleep Quality Index (PSQI)^65^	RCT^20^,^21^	MCID: 4.4 points (out of 21) [50 patients who underwent rotator cuff repair]^66^
	—	Sleep Timing and Sleep Quality Screening Questionnaire (STSQS)^67^	Observational^68^	Validated in cohort of 87 patients with biopsy-proven cirrhosis^69^ No reported MCID or MDC
	—	Basic Nordic Sleep Questionnaire (BNSQ)^70^	Observational^71^	No reported MCID or MDC
	—	PROMIS Sleep Disturbance^72^	Observational^42^	MCID: 3.5 points [186 surgical patients with adult spinal deformity]^73^ or 6.5 points [231 adults undergoing lumbar spine surgery]^74^ (out of 100)
	—	VAS^75^	RCT^22^	MCID: 10 mm (out of 100) [428 patients with insomnia aged 55 years or older]^75^
	Daytime sleepiness	Epworth Sleepiness Scale (ESS)^76^	RCT^20^,^22^	MCID: 2 points (out of 24) [639 patients with obstructive sleep apnea]^77^
Depression and Anxiety	Presence and burden of depressive and/or anxious symptoms	Cirrhosis-specific screening nomogram for depression^78^ and anxiety^79^	Observational^78^,^79^	No reported MCID or MDC
	—	Patient Health Questionnaire (PHQ)-9^80^	Observational^81^	MCID: 5 points (out of 27) [434 patients with late-life depression]^82^
	—	PHQ-4^83^	None	No reported MCID or MDC
	—	Generalized Anxiety Disorder-7 (GAD-7)^84^	Observational^81^	MCID: 4 points (out of 21) [261 patients with chronic depression]^85^
	—	Hospital Anxiety and Depression Scale (HADS)^86^	Observational^87^	MCID: 1.5 points [88 patients with chronic obstructive pulmonary disease]^88^ or 1.7 points [591 patients with cardiovascular disease]^89^ (out of 21)
Dyspnea and Breathlessness	Burden of breathlessness	Modified Medical Research Council (mMRC) Dyspnea Scale^90^	Observational^91^	MCID: 0.4 points (out 4) [238 patients with idiopathic pulmonary fibrosis]^92^
	—	VAS^93^	None	MCID: 10 mm change (out of 100 mm) [283 patients with chronic breathlessness]^93^
Nausea and Vomiting	Presence and severity of nausea and vomiting	Functional Living Index-Emesis^94^	None	No reported MCID or MDC
	—	PROMIS-Gastrointestinal Symptoms (Nausea Scale)^95^	None	No reported MCID or MDC
Multiple Symptoms	Burden and severity of multiple symptoms	Edmonton Symptom Assessment Scale (ESAS)^96^,^97^	Observational^98^	MCID (each symptom subscale): 1 point (out of 10) [796 patients with cancer]^99^ MCID (total symptom distress scale): 3–4 points (out of 90) [796 patients with cancer]^100^
	—	Memorial Symptom Assessment Scale (MSAS)^101^	Observational^102^	No reported MCID or MDC

Abbreviations: VAS, Visual analog scale; MCID: Minimal clinical important difference; MDC, minimal dectectable change, RCT: Randomized controlled trial, OBS: Observational studies.

#### Phase 4

Authors were then asked to comment on the draft Table 4, which was then finalized. The core writing group summarized the key references and, where available, the method and population used to establish the minimal detectable change (MDC) or minimal clinically important difference (MCID) of the selected symptom measures.

#### Phase 5

Authors were asked to provide research priorities for each of the symptoms listed in Tables 3–4. This would form the basis of Table 5 (future agenda for improving symptom management science in cirrhosis).

**TABLE 5:** Future agenda for improving symptom management science in cirrhosis

Symptoms	Clinical Gaps	Research Priorities
Muscle Cramps	(1) Limited comparative effectiveness and safety data on available, RCT-tested therapies (2) No inquiry into prophylactic treatments or behavioral treatments	(1) More long-term safety and comparative effectiveness data on available treatments (quinidine, taurine, pickle juice) (2) Development of behavioral and/or prophylactic strategies for muscle cramps
Pruritus	(1) Limited comparative effectiveness data on available, RCT-tested therapies (2) Limited evidence for behavioral treatments	(1) More long-term safety and comparative effectiveness data on available treatments (cholesytramine, colesevelam, gabapentin, rifampin, naltrexone, sertraline, and bezafibrate) (2) Testing of behavioral strategies for pruritus
Pain (Nociceptive, neuropathic, nociplastic)	(1) Difficulties in distinguishing various pain disorders (2) Limited safety data on effective pharmacologic treatments in cirrhosis (3) General lack of data on behavioral treatments in cirrhosis	(1) Further validation of pain scales in cirrhosis population (2) More long-term safety data on available pharmacologic agents. (3) Testing of behavioral strategies for various pain disorders.
Fatigue	(1) Lack of any effective pharmacologic treatments in cirrhosis (2) Limited data on behavioral treatments in cirrhosis	(1) Further validation of pain scales in the cirrhosis population (as opposed to PBC/PSC) (2) Further testing of new pharmacologic and behavioral treatments for fatigue.
Sexual dysfunction	(1) Discomfort among patients and providers for bringing up the topic (2) Limited tools for clinically assessing and addressing root causes of sexual dysfunction (including specific disorders) (3) General lack of effective pharmacologic and behavioral treatments in cirrhosis	1. Development and validation of symptom questionnaires tailored to patients with cirrhosis and that allow for an understanding of the pathophysiologic basis of sexual dysfunction. (2) Development and testing of pharmacologic and behavioral treatments
Sleep disorders	(1) Difficulty in clinically distinguishing HE from primary sleep disorders (2) Limited inquiry into the pathophysiologic basis for specific sleep disorders (3) Limited safety data on effective treatments for sleep disturbance	(1) Better diagnostic tools for distinguishing sleep disorders from HE (2) Further validation of sleep scales for use in cirrhosis. (3) More detailed guidance for utilizing polysomnography to understand the pathophysiologic basis of sleep disturbance. (4) Further testing of the safety and effectiveness of pharmacologic and behavioral strategies that can address underlying sleep disturbance.
Depression	(1) Limited safety data on effective treatments	(1) Further validation of depression symptom scales for use in cirrhosis. (2) Further testing of safety and effectiveness of pharmacologic and behavioral treatments.
Anxiety	(1) Limited safety data on effective treatments	(1) Further validation of anxiety symptom scales for use in cirrhosis. (2) Further testing of safety and effectiveness of pharmacologic and behavioral treatments.
Dyspnea/Breathlessness	(1) General lack of effective pharmacologic and behavioral treatments in cirrhosis	(1) Further validation of dyspnea and breathlessness measures in cirrhosis. (2) Further testing of safety and effectiveness of pharmacologic and behavioral treatments.
Nausea/Vomiting	(1) Limited safety data on effective treatments	(1) Further testing of safety and effectiveness of pharmacologic and behavioral treatments.

#### Phase 6

The core writing group then completed the manuscript. Each element was reviewed once more by each of the expert authors.

## RESULTS

### Clinical trial reporting

Essential features of the population, design, and results must be reported in a fashion that allows for a complete representation of the trial activities, facilitating the accurate interpretation of results by clinicians and patients. The use of well-recognized guidance with checklists (such as CONSORT) is highly encouraged.^103^ While many features enumerated in Table 1 are conventional elements of trial reporting, we highlight multiple considerations that are crucial for symptom-focused clinical trials. Population sample characteristics should include comprehensive details regarding disease severity, comorbidities (ie, substance use disorder), functional status, the use of psychoactive medications, and adjunctive therapies used to address the symptom under study (which may include the treatment of underlying secondary causes). With regards to study results, the reporting of treatment response should include absolute differences as well as the proportion of patients who achieved complete response or MCIDs. Competing risks or events should be explicitly addressed in both design and analysis.

### Common study design features and their requirements

Table 2 specifies multiple key study design features. Efforts should be made to ensure appropriate representation of the target population with respect to age, sex, race, ethnicity, and socioeconomic status.^104^,^105^ Inclusion criteria should specify a minimum symptom burden (using a consistent rating system) to standardize the study sample. The selection of comparators should ensure comparison to the highest standard of “usual care,” and “usual care” should be pre-specified at the study outset. Therapy dose and frequency should be selected deliberately based on known safety and pharmacokinetic data. Postprotocol and rescue therapies, as well as safety monitoring, should be specified at the outset. Pilot trials should focus on feasibility outcomes (eg, the ability to deliver intervention), designating therapeutic effects as exploratory. Follow-up durations should be sufficient to assess therapeutic effectiveness using validated instruments while also considering the short life expectancy for many patients with cirrhosis. For example, a trial of a selective serotonin reuptake inhibitor should follow patients for at least several weeks based on the pharmacotherapeutic properties of the medications. Similarly, if a medication is used for a short duration, the follow-up time should be built into the study timeline to allow for detection of medication withdrawal as appropriate or symptom return.

### Endpoint assessment

Endpoints for symptom-focused clinical trials may include objective metrics that are assessed by clinicians (eg, polysomnography) and/or patient-reported outcomes (PROs), which can include instruments such as patient diaries (eg, sleep diary), numerical rating or visual analog scales (VAS), or symptom questionnaires (eg, Epworth Sleepiness Scale). While objective metrics may remain the gold standard for symptom assessment in clinical trials (eg, polysomnography for measuring sleep), their use is limited to phenomena that can be directly observed or measured. In addition, objectively measured assessments can be cumbersome to integrate into the research settings, limiting their feasibility for use in large-scale clinical trials. PRO instruments, in addition to their ease of administration, have become important outcomes used to support clinical, health policy, and regulatory decision-making. For the US Food and Drug Administration, PROs can be used to support labelling claims for medical devices and pharmaceuticals.^106^,^107^ Despite their many potential applications, standardized, well-validated PROs in the cirrhosis population are lacking.

For symptom-focused clinical trials using a PRO instrument to assess the primary outcome, investigators should critically examine whether the PRO instrument is “fit for purpose” 108 for patients with cirrhosis.^107^ More specifically, does the PRO instrument have evidence of content validity to capture clinically meaningful improvements in symptoms both validly and reliably among patients with cirrhosis?^109^ Investigators can assess a PRO instrument’s content validity through a careful review of the original studies describing its development. Importantly, a key aspect of assessing whether a PRO instrument is fit for purpose is whether the population in which the PRO development and validation studies were conducted is consistent with the target population for the clinical trial. For example, PRO measuring pruritus symptoms that were developed in the dialysis population may not adequately capture the experience of patients with primary biliary cholangitis. For PROs developed specifically for the cirrhosis population, investigators should assess if the patients in the initial validation studies had different disease etiology (eg, primary biliary cholangitis versus NAFLD) or severity (eg, compensated cirrhosis versus decompensated cirrhosis) to the target population for the clinical trial. If there are significant differences between the population in which the PRO validation work was performed and the target population for the clinical trial, investigators should provide evidence justifying the selection of the PRO instrument for the new population.^107^ This may require qualitative methods to confirm content validity of the existing PRO in the new study population though focus groups or one-on-one interviews, specifically assessing its relevance, comprehensibility, and comprehensiveness.^110^

In addition to content validity, investigators should assess construct validity (does the PRO measure what it was intended to measure?), reliability (does the PRO produce the same outcome on repeated assessments when there has not been a change in status?), and responsiveness (does the PRO detect differences within or between patients over time in response to a change in status?).^107^,^109^ The psychometric properties of a PRO can be assessed through a review of its original development and subsequent validation studies. Useful frameworks to systematically review the validity and other psychometric properties of available PRO instruments include the Consensus-based Standards for the selection of health Measurement Instruments (COSMIN) guidelines^109^,^111^,^112^ and the FDA recommendations for PRO development.^107^

Before undertaking a symptom-focused clinical trial, investigators should carefully evaluate the methods that were used to establish the reported MDC for the PRO instrument of choice. The MDC for a PRO instrument can be established using anchor-based and/or distribution-based approaches. MDC values determined using anchor-based methods (such as the Global Rating of Change^113^) establish the MCID for the PRO instrument.^114^ MDC values established using distribution-based approaches, such as the SE of measurement, may identify differences in PRO scores that are statistically significant but may not be clinically meaningful or important to patients. The FDA recommends using anchor-based approaches to estimate the MDC for PRO instruments, with distribution-based methods providing only supportive, rather than primary, data to justify the MDC.^107^ For PRO instruments that have an MDC determined using distribution-based approaches, it is recommended that investigators incorporate the use of an anchor in their clinical trial to determine the MCID of the instrument in their study.^115^ If an MDC has not yet been defined for the PRO of interest, investigators can consider the following approaches: (1) reviewing previous symptom management clinical trial literature to identify evidence for the MDC for the PRO; (2) using a combination of anchor-based and distribution-based approaches to estimate the MDC from a pilot randomized control trial or longitudinal observational study; and/or 3) determining the MDC through expert consensus using Delphi methods.^116^

### Key symptom targets

Patients with cirrhosis experience a vast array of symptoms for which more evidence is needed to inform best practices. In Table 3, we provide the set of symptoms targeted by the panel, with brief explanations and summaries of prior clinical trial-tested and standard-of-care interventions. In Table 4, we detail how each symptom target can be assessed using a consensus set of symptom-focused instruments, any prior use in patients with cirrhosis, and further explanation of their psychometric properties. This summary of prior validation work should serve as a guide to clinical trialists interested in using these instruments in future studies. Trialists should note that many of these instruments have not been evaluated for content validity, specifically among patients with cirrhosis. Further, MCIDs unique to the cirrhosis population have not been developed for any of the instruments, which should be viewed as a limitation. Future instrument development may be needed to close these gaps in psychometric validation.

### Muscle cramps

Muscle cramps are prevalent in more than 50% of patients with cirrhosis 3 and are related to alterations in skeletal muscle metabolism, nerve function, and plasma volume. Pickle juice has been shown to reduce cramp severity, while quinidine and taurine have been shown to reduce cramp severity and frequency.^6^,^8^,^9^ Treatments are often ineffective, and even treatment responders have persistent unmet needs. Based on their use in cramp-focused RCTs, it is recommended that cramps be assessed using a VAS for cramp severity, cramps per week, and cramp duration in minutes.

### Pruritus

Itch frequently complicates cirrhosis, particularly for those with biliary etiologies but also for those with non-biliary diseases. The pathophysiology is likely heterogeneous, including excessive or dysregulated circulating bile acids, lysophosphatidic acid, and other yet-to-be isolated pruritogens.^117^ Most clinical trials have enrolled patients with the biliary disease and have demonstrated efficacy for therapies that address multiple targets in the itch development and sensory pathways, including bile acid binders, rifampin, fibrates, naltrexone, gabapentin, or sertraline.^118^ Our expert panel suggested using the 5D-Pruritus scale to capture the degree, duration, direction, distribution, and disability associated with itching.^27^ Alternatively, pruritus intensity can be captured using a VAS.^24^

### Pain

Chronic pain can be characterized using 3 mechanistic descriptors: nociceptive, neuropathic, and nociplastic.^119^ Nociceptive pain is common in cirrhosis—for example, fractures,^120^ abdominal distension,^121^ muscle cramping^2^—and is caused by ongoing tissue damage. Neuropathic pain is also common owing to highly prevalent alcohol use disorder, diabetes, and cirrhosis-related metabolic disorders.^122^,^123^ It is caused by nerve damage or disease in the peripheral or central nervous system (CNS).^119^ In contrast, the term ‘nociplastic pain’ was introduced in 2016 to describe pain with no evidence of tissue or nerve damage but with “clinical and psychophysical findings that suggest altered nociception” 119,124 such as pain associated with fibromyalgia or irritable bowel syndrome.^124^,^125^ Patients with nociplastic pain may present with widespread pain refractory to intervention (eg, opioids),^126^ as well as accompanying CNS-driven complaints (eg, fatigue, sleep difficulty, mood dysregulation, and memory problems).^127^,^128^ The putative mechanism of nociplastic pain is ‘central sensitization,’ characterized by aberrant pain processing in the peripheral and central nervous system that leads to increased pain sensitivity,^119^,^125^,^129^ augmented pain processing, and diminished pain inhibition.^119^,^125^ For the purpose of effective treatment and clinical trial design, it is important that the patient’s pain phenotype is rigorously defined. Specific causes of nociceptive pain—for example, cramps and ascites—should be assessed using measures validated for those conditions. Generic measures for acute pain include the Brief Pain Inventory for severity and interference;^23^,^31^ generic measures for chronic pain should include the PEG scale (a composite of VAS for pain interference and intensity for pain, enjoyment of life, and general activity),^23^,^34^ McGill Pain Questionnaire,^39^ Edmonton Symptom Assessment Score,^96^ or the Pain Disability Index.^36^,^130^ PROMIS measures for pain interference can also be used.^41^ Neuropathic pain and contributions to pain should be defined using PainDETECT,^45^ Neuropathic Pain Symptom Inventory,^46^ or Neuropathic Pain Scale.^47^ Nociplastic pain can be defined and tracked using the Fibromyalgia Survey Questionnaire,^48^ which contains 2 subscales, the Wide-spread Pain Index and the Symptom Severity Score, with items related to fatigue, unrefreshed sleep, cognitive problems, headache, abdominal pain, and depression.^131^ These subscales can be used to diagnose fibromyalgia and quantify “central sensitization” for any pain complex.^131^ There have been no tested treatments for pain among patients with cirrhosis.

### Fatigue

Fatigue is a common and vexing symptom that frequently complicates chronic illness and particularly so for cirrhosis. Many instruments for the assessment of health-related quality of life and symptom interference include measures for fatigue. To explicitly assess the impact of an intervention on the severity of fatigue, it is important to use fatigue-specific scales. The Fisk Fatigue Severity Score,^49^,^52^,^132^ Multidimensional Fatigue Inventory,^17^,^54^ and the PROMIS-fatigue short form^56^ were selected by the panel. Interventions to improve fatigue have focused on improving the underlying liver disease or addressing the symptom directly. For example, HE can cause fatigue, and HE-directed therapies can improve fatigue.^133^,^134^ Patients with fatigue complicating PBC have been tested with fluvoxamine and modafinil, but neither was found to be effective.^17^,^18^

### Sexual dysfunction

Sexual dysfunction is estimated to affect over half of men and women with cirrhosis.^135^,^136^ Hypogonadism, high estrogen-to-testosterone ratio, medication side effects (eg, aldosterone-antagonists, beta-blockers), autonomic neuropathy, and comorbid mental and physical health limitations all contribute. Erectile dysfunction can be successfully treated with phosphodiesterase inhibitors.^19^ However, their utility may be limited in the context of decompensated cirrhosis. Moreover, there are vast unmet needs with respect to women’s sexual function and reduced libido. The panel has selected 4 tools to measure sexual function: the International Index of Erectile Function,^57^ the Female Sexual Function Index,^59^ the Arizona Sexual Experience Scale,^62^ or PROMIS Sexual Function and Satisfaction Measures Version 2.0,^64^ which can be used for both sexes.

### Sleep Disorders

Sleep disturbances, such as excessive daytime sleepiness and insomnia, affect over half of the patients with cirrhosis, even among patients without HE.^135^ The presence of ascites, volume overload, and pruritus may contribute to poor sleep as well. Objective measures of sleep quality, such as polysomnography and actigraphy, are useful clinical tools that can also be used as outcome measures in clinical trials. However, many other PROs have been developed for sleep disturbances that can be easily measured and monitored. The panel selected the Pittsburgh Sleep Quality Index,^65^,^137^ Sleep Timing and Sleep Quality Screening Questionnaire,^67^,^138^ Basic Nordic Sleep Questionnaire,^70^ PROMIS Sleep Disturbance,^72^,^139^ VAS,^75^ and Epworth Sleepiness Scale 76,140 as instruments that should be considered. The Sleep Timing and Sleep Quality Screening Questionnaire is unique in that it was validated specifically for patients with cirrhosis.^69^

### Depression and anxiety

Moderate to severe depression affects nearly 1 in 6 individuals with cirrhosis, while moderate to severe anxiety affects nearly half.^141^ Both conditions are debilitating, comorbid with multiple other physical complaints, and associated with increased mortality.^142^ Organic causes, such as vitamin deficiencies, HE, and dementia, should be considered and treated when establishing the diagnosis.^1^ The Patient Health Questionnaire-9^80^ and its short-form,^83^ as well as the Generalized Anxiety Disorder-7^84^ Scale, have been extensively validated in primary care populations and are recommended to screen patients with cirrhosis for these conditions. The Hospital Anxiety and Depression Scale was developed for and is useful to assess hospitalized patients, including those with cirrhosis.^86^ Recently, nomograms have been developed to screen for anxiety and depression in patients with cirrhosis.^78^,^79^ While validation remains to be completed for these tools, they are the only measures that have been specifically developed for this population. There have been no tested treatments for depression and anxiety among patients with cirrhosis.

### Dyspnea/Breathlessness

Breathlessness in patients with cirrhosis may be due to volume overload or primary pulmonary causes, including hepatopulmonary syndrome, portopulmonary syndrome, and other chronic disorders.^1^ Addressing underlying secondary causes should be first considered before primary treatment. Scales that have been used to track dyspnea and breathlessness for clinical trials include the Modified Medical Research Council Dyspnea Scale 90,92 and the VAS,^93^ which were selected by the panel. There have been no tested treatments for dyspnea/breathlessness among patients with cirrhosis.

### Nausea/Vomiting

Multiple etiologies, including medications, adrenal insufficiency, electrolyte imbalance, and constipation, may contribute to nausea, which commonly affects patients with cirrhosis.^98^,^143^ Limited safety data exist for multiple antiemetic therapies.^1^ Scales that have been developed for nausea and vomiting, selected by the panel, include The Functional Living Index-Emesis^94^ and PROMIS-Gastrointestinal Symptoms.^95^,^144^ There have been no tested treatments for nausea/vomiting among patients with cirrhosis.

In addition to instruments used for single symptoms, the Edmonton Symptom Assessment System^96^ and Memorial Symptom Assessment Scale^97^,^101^ are well-validated instruments that can be used to capture multiple symptoms at once.

### Future agenda for improving symptom management science in cirrhosis

Advancing the field of symptom management in cirrhosis requires an appreciation for both the pressing clinical and scientific gaps. The panel identified 3 primary needs in the field. The first is to ensure that costly and time-consuming clinical trials follow rigorous study design recommendations. The second is a need to select and implement standard measures and ensure their validation over time in this unique population. In particular, validated instruments should ideally have anchor-based MCIDs that can track improvements over time. Very few of these currently exist for the cirrhosis population. Investigators should consider the use of PROMIS measures, which were developed to be comparable across populations and studies.^145^ Given the number of complex, intertwined symptoms in patients with cirrhosis and their multifactorial causes, it is particularly important to tease apart the mechanistic effects of interventions using standard measures. Finally, there is a paucity of evidence supporting interventions in this population. To date, there have been no tested treatments for pain, depression, anxiety, breathlessness/dyspnea, nausea, or vomiting among patients with cirrhosis.

While there are a number of pharmacologic treatments used for these conditions in other populations, many require further testing for safety and comparative effectiveness in people with cirrhosis. Likewise, testing non-pharmacologic symptom management strategies is a promising but resource-intense and complex endeavor. However, such interventions also have the potential to address multiple symptoms simultaneously. While all symptoms should ideally have non-pharmacologic options rigorously appraised, the group placed the greatest priority on developing behavioral interventions for symptoms lacking safe pharmacologic options. For symptoms that have more validated measures and tested pharmacologic treatments (ie, muscle cramps, pruritus, and pain), the next step is to assess the safety and comparative effectiveness of the pharmacotherapies and non-pharmacotherapies. Developing an infrastructure for this field of science will require considerable time, cost, and resources; thus, federal and nonfederal agencies should expeditiously prioritize funding in this area of research, given the immense clinical need, limited data, and a high benefit for improving the quality of life of this vulnerable population.

## CONCLUSION

We developed this guidance to support clinical trialists in crafting high-quality interventions that address the symptom burden faced by patients with cirrhosis. From our review, it is clear that gaps in measurement, safety data, and comparative effectiveness information remain for the most common symptoms. We hope that the timely appreciation of PRO research in this population will help drive innovation and advance the clinical care of this population, which deserves not just a longer life, but a better quality of life.
